# The genetic variation rs6903956 in the novel androgen-dependent tissue factor pathway inhibitor regulating protein (*ADTRP*) gene is not associated with levels of plasma coagulation factors in the Singaporean Chinese

**DOI:** 10.1186/s12959-016-0124-y

**Published:** 2017-01-07

**Authors:** Xuling Chang, Hui-Lin Chin, Swee-Chye Quek, Daniel Y. T. Goh, Rajkumar Dorajoo, Yechiel Friedlander, Chew-Kiat Heng

**Affiliations:** 1 Department of Paediatrics, Yong Loo Lin School of Medicine, National University of Singapore, NUHS Tower Block, Level 12, 1E Kent Ridge Road, Singapore, 119228 Singapore; 2 Khoo Teck Puat - National University Children’s Medical Institute, National University Health System, Singapore, Singapore; 3 Genome Institute of Singapore, Agency for Science, Technology and Research, Singapore, Singapore; 4 School of Public Health and Community Medicine, Hebrew University of Jerusalem, Jerusalem, Israel

**Keywords:** Coagulation factors, Coronary artery disease, Androgen-dependent tissue factor pathway inhibitor regulating protein, Genetic variant

## Abstract

**Background:**

Genome-wide association study (GWAS) has reported that rs6903956 within the first intron of androgen-dependent tissue factor pathway inhibitor (TFPI) regulating protein (*ADTRP*) gene is associated with coronary artery disease (CAD) risk in the Chinese population. Although ADTRP is believed to be involved in the upregulation of TFPI, the underlying mechanism involved is largely unknown. This study investigated the association of rs6903956 with plasma Factor VII coagulant activity (FVIIc) and fibrinogen levels, which are regulated by TFPI and are independent risk predictors for CAD.

**Methods:**

We conducted the analysis in both Chinese adult (*N* = 309) and neonatal cohorts (*N* = 447). The genotypes of the rs6903956 single nucleotide polymorphism (SNP) were determined by the polymerase chain reaction restriction fragment length polymorphism method (PCR–RFLP). FVIIc and fibrinogen level were measured from citrated plasma. The association between rs6903956 and coagulation factors was tested by linear regression with adjustment for possible confounders. Analysis was carried out in adults and neonates separately.

**Results:**

No significant association was observed between rs6903956 and plasma FVIIc nor fibrinogen levels with adjustment for age, gender, body mass index (BMI) and cigarette smoking in adults (P for FVIIc = 0.464; P for fibrinogen = 0.349). The SNP was also not associated with these two coagulation factors in the neonates (P for FVIIc = 0.579; P for fibrinogen = 0.359) after adjusting for gestational age, gender and birth weight.

**Conclusions:**

SNP rs6903956 on *ADTRP* gene was not associated with plasma FVIIc nor fibrinogen levels.

## Background

Coronary Artery Disease (CAD) is one of the most important causes of mortality and morbidity in both developed and developing countries [1], with men having higher disease risk than women of a given age [2, 3]. In 2013, it resulted in 8.14 million deaths (16.8%) compared to 5.74 million deaths (12%) in 1990 [4]. CAD occurs when atherosclerosis develops and plaques form to narrow and block the coronary arteries. Patients may not be aware of it for decades until a sudden heart attack finally arises, which brings a heavy disease burden to the public health system and the individuals. CAD is a complex disease and its susceptibility is contributed by both genetic and lifestyle factors.

With the recent rapid development of genotyping technologies, genome-wide association studies (GWAS) have increasingly been used to identify susceptibility loci for complex diseases. Several novel susceptibility loci have also been reported for CAD thus far, including rs6903956 in the *C6orf105* region of chromosome 6p24.1, which was specifically identified in the Chinese population. The minor risk allele A of rs6903956 was found to be associated with reduced mRNA expression of *C6orf105* and increased risk of CAD [5]. This region was later found to code for a novel membrane protein which could regulate mRNA expression, cellular distribution and anticoagulant activity of tissue factor pathway inhibitor (TFPI), both in native conditions and in response to androgen. Thus the novel protein has been named androgen-dependent TFPI regulating protein (ADTRP). It was further reported that a decreased expression of ADTRP resulted in the reduction of mRNA expression of TFPI. Therefore, ADTRP is postulated to have an anti-coagulating effect [6].

TFPI is a known inhibitor of coagulation, as it reversibly inhibits Factor Xa (FXa) and also tissue factor-factor VIIa (TF-FVIIa) complex in the presence of FXa. TF-FVIIa complex is the main initiator of the extrinsic coagulation cascade in cardiovascular disease [7]. Tissue factor (TF) is normally found on the outside of blood vessels and not exposed to the blood. Once injury occurs, TF is exposed to the bloodstream and binds to circulating Factor VII (FVII) [8, 9]. FVII will be then rapidly converted to the activated form (FVIIa), which binds factor X (FX) and results in its conversion to activated FX (FXa), leading eventually to thrombin activation. Thrombin converts soluble fibrinogen to insoluble fibrin strands and form blood clots [9–11]. Previous studies have shown that coagulation factors have an essential role in advancing atherosclerosis and CAD [12–14]. High levels of FVII coagulant activity (FVIIc) and fibrinogen have been found to be significantly associated with increased risk of coronary events [15].

Previously, the association between rs6903956 within the first intron of *ADTRP* and risk for CAD was replicated in our Singaporean Chinese population [16]. The study also found that the association was not mediated through plasma lipid levels. As ADTRP has been found to up-regulate TFPI, we hypothesized that it is likely to have an impact on key coagulation factors, such as FVIIc and fibrinogen, which are known to have an association with CAD risk. The effect of the genetic variant in *ADTRP* on CAD might thus be due to its impact on plasma FVII and fibrinogen levels. This study was conducted to investigate the effect of the rs6903956 variant on FVIIc and fibrinogen levels in both Singaporean Chinese adults and neonates.

## Methods

### Study population

To determine the impact of the *ADTRP* genetic variant on plasma FVIIc and fibrinogen, we conducted the analysis in a group of healthy subjects who were free from CAD at the time of recruitment. In total, 421 adult subjects were included in the analysis. The participants were volunteers who underwent regular medical examination at a health screening center in the community. The participants were excluded from the study if they suffered from (1) hypertension, (2) CAD, (3) diabetes, (4) had abnormal ECG. All the study subjects were required to fill in a questionnaire which included the following information: age, height, weight, race, cigarette smoking history, oral contraceptives usage, medical history and family history. Body mass index (BMI) was calculated as weight in kilograms (kg) divided by height in meter square (m^2^).

The neonatal subjects involved in our study were recruited from consecutive healthy Chinese newborn babies who were delivered at the National University Hospital in Singapore. The following groups of subjects were excluded from the analysis: (1) babies of mixed heritage; (2) parents with vague information on family or medical history; (3) premature babies with less than 37 weeks of gestational age or less than 2500 grams of birth weight; (4) babies who needed medical treatment or observation in the neonatal ward for had perinatal problems, and those with major congenital deformities, or known or suspected chromosomal syndromes; (5) mothers with recognized medical problems before pregnancy, or gestational conditions such as diabetes or pregnancy-induced hypertension [17]. A total of 1267 neonates were recruited from the three major ethnic groups in Singapore and of these, 447 of Chinese ethnicity with available FVIIc, fibrinogen and rs6903956 genotype information were included in our analysis.

### Blood collection

Details of the blood collection procedures were reported previously for the adult [18] and neonatal populations [19]. Briefly, blood samples for the adult study were collected after subjects had fasted overnight for at least 10 h while the neonatal samples were obtained from umbilical cords. Plasma was separated within 3 h from blood collection tubes that were anti-coagulated with sodium citrate. The plasma was aliquoted into three cryotubes, immediately snap-frozen in liquid nitrogen and stored at -70 °C until use. Measurements of coagulant levels were performed within 4 weeks of storage. Packed cells were stored at -20 °C for the purpose of extraction of genomic DNA.

### Measurement of coagulation factors

FVIIc was estimated by a one-stage semiautomatic bioassay in an H Amelung coagulometer at 37 °C [20]. FVIIc can activate FX in the presence of tissue thromboplastin and calcium. FVII deficient plasma will have a greatly prolonged prothrombin time (PT). When FVII deficient plasma were mixed with the test plasma, the degree of correction of the PT is proportional to the level of plasma FVII of the participants. The intra-assay and inter-assay coefficients of variation of FVIIc were 1.83 and 6.21% respectively.

Fibrinogen was measured using the von Clauss assay [21] in the H Amelung coagulometer. Soluble fibrinogen, in the presence of excess bovine thrombin, can be converted into insoluble fibrin and a clot is formed. The clotting time obtained is inversely proportional to the concentration of fibrinogen. The intra-assay and inter-assay coefficients of variation of fibrinogen were 3.0 and 4.8% respectively.

### Genotype determination

Genotype was determined by the polymerase chain reaction restriction fragment length polymorphism method (PCR–RFLP). A 409 base pairs (bp) amplicon of the *ADTRP* gene was amplified from genomic DNA. The DNA sequence of the *ADTRP* was obtained from National Center for Biotechnology Information (NCBI) dbSNP database (build 37). Detailed information regarding the primer design, optimization, condition of amplification reactions, PCR products digestion, incubation conditions for RFLP and confirmation of the genotyping results was described elsewhere [16]. The presence of 2 DNA fragments with the length of 378 and 31 bp respectively indicated the presence of the minor risk A allele while a single band of 409 bp indicated the presence of the major G allele. A total of 6 randomly selected samples including two samples each for the three different genotypes were confirmed by Sanger sequencing. Additionally, 20 random samples were also sequenced and all 26 samples had 100% concordance for genotypes obtained from both methods.

### Statistical analysis

Among all the study subjects recruited, 309 adults and 447 neonates had complete information for both genotypes and coagulation factor measurements, thus data from these participants were included in the analysis.

Quantitative variables were presented as mean ± SD (standard deviation) while categorical variables were presented as number of individuals (percentage %). Analysis was carried out in adults and neonates separately. FVIIc and fibrinogen levels were not normally distributed in both adults and neonates. Hence, they were both Z-score transformed for subsequent analysis. Pearson’s *χ* ^2^ test was also used to check whether genotype frequencies significantly departed from Hardy–Weinberg expectations (HWE). Considering the minor allele frequency (MAF) of rs6903956 and the sample size of the cohorts, to achieve a power above 80%, the effect size should be at least 0.52 and 0.39 for adult and neonates respectively. Linear regression was used to investigate the association between rs6903956 and Z-score transformed coagulation factors. In adults, age, BMI, gender and cigarette smoking status were included into the model as covariates while in neonates, gender, gestational age and birth weight were included as covariates. All statistical analyses were carried out using STATA 12.0 (Stata Corp, College station, TX) and a 5% type I error was set to indicate statistical significance (two-tailed) in all analyses.

## Results

The main demographic and clinical characteristics of adult and neonatal study subjects are summarized in Table 1.

**Table 1:** Descriptive characteristics of study subjects

	Adults (*N* = 309)	Neonates (*N* = 447)
Age (years)	34.95 ± 12.34	-
Gestational age (weeks)	-	39.15 ± 1.21
BMI (kg/m^2^)	22.16 ± 3.55	-
Birth weight (g)	-	3253.05 ± 355.33
Gender (Male %)	154 (49.84%)	215 (48.10%)
FVIIc (%)	114.28 ± 36.53	59.73 ± 18.31
Fibrinogen (mg/dl)	276.45 ± 64.78	128.73 ± 53.40
Cigarette Smoking		
Never	258 (84.04%)	-
Ever	4 (1.30%)	-
Current	45 (14.66%)	-
rs6903956		
GG	278 (89.97%)	390 (87.25%)
GA	31 (10.03%)	57 (12.75%)

Data presented as Mean ± Standard Deviation (SD) or N (%)

The observed genotype frequencies of rs6903956 did not depart significantly from HWE in both adults (*p* = 0.353) and neonates (*p* = 0.150). The homozygous genotype for the minor allele A was absent in both cohorts. The minor allele frequency (MAF) of rs6903956 was 5.0 and 6.4% respectively for adults and neonates, which is similar to the MAF previously reported in Han Chinese and Japanese population [5].

Table 2 shows the association between coagulation factors and rs6903956 on *ADTRP* in adult and neonate population. In adults, levels of FVIIc and fibrinogen were not found to be significantly impacted by rs6903956 after adjusting for the confounding effects of age, gender, BMI and cigarette smoking status (P for FVIIc = 0.464; P for fibrinogen = 0.349). The association was also not significant in the neonates after adjusting for gestational age, gender and birth weight (P for FVIIc = 0.579; P for fibrinogen = 0.359).

**Table 2:** Genotypic coagulation factors levels of rs6903956 in the Chinese adults and neonates population

	Adults			Neonates		
	GG (*N* = 278)	GA (*N* = 31)	P*	GG (*N* = 390)	GA (*N* = 57)	P*
FVIIc	108.60 (36.00, 226.00)	108.00 (46.00, 160.00)	0.464	55.65 (25.99, 184.70)	54.24 (31.40, 150.70)	0.579
Fibrinogen	266.35 (4.00, 584.26)	274.00 (62.44, 434.92)	0.349	132.53 (43.49, 260.30)	147.80 (46.11, 271.70)	0.359

Data presented as Median (Minimum, Maximum)

*The association between genotype and z-score transformed coagulation factors were tested by linear regression with adjustment for age, gender BMI and cigarette smoking in adults; and gestational age, gender and birth weight in neonates

## Discussion

The formation of arterial thrombi induced by ruptured atherosclerotic plaques plays a central role in the development of CAD [22]. The exposure of a thrombogenic surface in the ruptured plaque triggers platelet activation and coagulation, which further promotes thrombosis in the coronary arteries. The coagulation system consists of a series of procoagulant and anticoagulant proteins which are present in circulation [23]. In vivo, the exposure of TF to the circulating blood initiates activation of the extrinsic coagulation cascade. It binds to FVII on the cell surface and the TF-FVII complex activates Factor IX (FIX) and FX. FXa converts prothrombin to thrombin and then thrombin activates platelets and converts fibrinogen to fibrin. FVII plays a central role in initiating the process of coagulation in conjunction with TF while fibrinogen is essential for forming the final fibrin clot [24, 25]. Both FVII and fibrinogen were reported to be independent risk predictors for CAD [24, 26–28]. Several anticoagulant systems operate at the same time of coagulation to regulate procoagulant activity [23]. One of the important anticoagulants is TFPI, which inactivates both FVIIa and FXa, thus inhibiting the extrinsic coagulation pathway [29].

In 2011, Wang et al. [5] first showed that rs6903956, which is located in intron 1 of the *C6orf105* region, on chromosome 6p24.1 was associated with CAD risk in Han Chinese population. The mRNA expression of *ADTRP* gene was significantly lower in individuals with AA and AG genotypes compared to those with GG genotypes. Subsequently, this region has been reported to encode a gene for a androgen-dependent protein that could regulate the mRNA expression, cellular distribution and anticoagulant activity of TFPI [6]. It is suggested that a decreased expression of *ADTRP* may lead to a reduction in TFPI expression and thus result in an increased risk for atherosclerosis and CAD. The association between rs6903956 and CAD risk has been replicated in independent Chinese cohorts [16, 30]. This single nucleotide polymorphism (SNP) was also reported to be associated with CAD in the Japanese, although the risk allele in their population was G and hence different from the Chinese [31]. This SNP has not been found to be associated with CAD in the European populations [32].

Previously, our group had replicated the association between rs6903956 and CAD risk in the Singaporean Chinese and found that plasma lipids such as high density lipoprotein (HDL) cholesterol, low density lipoprotein (LDL) cholesterol and triglycerides failed to explain the association [16]. Under the influence of androgen, ADTRP has been found to up-regulate TFPI [6]. This has downstream effects on FVII and fibrinogen, both of which are known to be associated with increased CAD risk [15]. In this study, we investigated whether there is any association between rs6903956 and the two coagulation factors. No significant association between the SNP and the two coagulation factors was observed in both the adults and neonatal subjects. We tested for the association in the neonatal cohort as we assumed that the effect of any genetic factors could be observable at birth. Our group had previously shown that variation of FVIIc levels at birth could be significantly determined by genetic factors such as polymorphisms in the FVII gene [17]. Hence, determination of the potential impact of the *ADTRP* variant was extended to the neonatal population. One advantage of studying genetic effects in the neonates is that the confounding effects of environmental factors could be minimized as these can be challenging to tease apart in adult studies.

As there is no prior report on the effect of the *ADTRP* genetic variants on coagulation factor levels, we are unable to estimate the sample size required for such a study to be adequately powered. The post analysis power estimates from this study is 15.12% for FVIIc and 28.16% for fibrinogen. Given the very small effect sizes observed in this study, a sufficiently large sample size to detect a statistically significant impact of rs6903956 on FVIIc and fibrinogen is estimated to be 7000. With such a large sample size required, it is unlikely that there is any notable clinical impact of this SNP on these two coagulation factors.

## Conclusion

In conclusion, we found that despite the *ADTRP* gene being involved in the regulation of TFPI, its genetic variant rs6903956 is not associated with plasma FVIIc and fibrinogen levels.

## Abbreviations

ADTRP: Androgen-dependent tissue factor pathway inhibitor regulating protein
bp: Base pairs
BMI: Body mass index
CAD: Coronary artery disease
FIX: Factor IX
FVII: Factor VII
FVIIc: Factor VII coagulant activity
FX: Factor X
FXa: Factor Xa
GWAS: Genome-wide association study
HWE: Hardy–Weinberg expectations
HDL: High density lipoprotein cholesterol
LDL: Low density lipoprotein
MAF: Minor allele frequency
NCBI: National Center for Biotechnology Information
PCR–RFLP: Polymerase chain reaction restriction fragment length polymorphism
PT: Prothrombin time
SNP: Single nucleotide polymorphism
SD: Standard deviation
TF: Tissue factor
TF-FVIIa: Tissue factor-factor VIIa
TFPI: Tissue factor pathway inhibitor
